# Considerations for tactile perceptual assessments: impact of arm dominance, nerve, location, and sex in young and older adults

**DOI:** 10.1007/s00221-025-07044-5

**Published:** 2025-03-15

**Authors:** Emily M. Tirrell, Nahid Kalantaryardebily, Anna C. Feldbush, Lindsey Sydnor, Christopher Grubb, Kevin Parcetich, Netta Gurari

**Affiliations:** 1 Translational Biology, Medicine, and Health Program, Virginia Polytechnic Institute and State University, Blacksburg, Virginia 24061 USA; 2 Department of Mechanical Engineering, Virginia Polytechnic Institute and State University, Blacksburg, Virginia 24061 USA; 3 School of Neuroscience, Virginia Polytechnic Institute and State University, Blacksburg, Virginia 24061 USA; 4 Center for Biostatistics and Health Data Science, Virginia Polytechnic Institute and State University, Roanoke, Virginia 24061 USA; 5 Department of Physical Therapy, Radford University, Roanoke, Virginia 24061 USA; 6 Department of Biomedical Engineering and Mechanics, Virginia Polytechnic Institute and State University, Blacksburg, Virginia 24061 USA

**Keywords:** Tactile perception, Age, Arm dominance, Location (elbow/hand), Nerve (median/ulnar/radial)

## Abstract

**Purpose:**

Intact tactile perception is essential to successfully interact with objects. While tactile examinations exist for capturing tactile impairments, recent investigations underscore that these examinations remain insufficient, particularly for adults following a neurological injury. To inform the design of improved tactile assessments, this study comprehensively captures factors that can influence tactile perception in young and older adults who are neurologically intact.

**Methods:**

We examined the impact of arm dominance (dominant/non-dominant), nerve (median/ulnar/radial), location (hand/elbow), and sex (male/female) on thresholds at which electrotactile stimuli could be consciously detected when applied to the skin in 20 young and 14 older right-arm dominant participants.

**Results:**

Significant differences depending on arm dominance were not found in young (p = 0.6781) or older (p = 0.2786) adults. Yet, the nerve tested did yield differing thresholds in young (p < 0.0001) and older (p < 0.0001) adults. In young adults, thresholds were less at the hand than elbow (p = 0.0031). In older adults, the average threshold was greater at the hand than elbow. Importantly, in older adults the threshold at the hand increased with age to a greater extent than at the elbow (p < 0.0001). Thresholds were greater in males than females in young adults (p = 0.0004), whereas no significant sex differences were observed in older adults (p = 0.2560).

**Conclusion:**

This work highlights the importance of addressing numerous factors and their interactions when assessing tactile perception (e.g., arm dominance, nerve, location, sex, age). Findings can inform the design of improved tactile assessments that more accurately capture why impairments arise, including following a neurological injury.

## Introduction

Intact tactile, or touch-related, perception is critical for accurately perceiving and interacting with physical stimuli. Whether hugging a loved one, typing on a keyboard, or holding a cup of coffee, the ability to perceive tactile stimuli greatly influences the successful performance of daily tasks Cole (1992); Bolognini et al. (2016). Various patient populations, including those living with a neurological injury, experience tactile impairments, with the severity of the tactile impairments corresponding to their movement recovery and physical function post injury Sullivan and Hedman (2008); Grefkes and Fink (2020). Despite this understanding of the importance of intact tactile perception, numerous reviews underscore that existing tactile assessments remain insufficient for reasons including poor reliability, inadequate validity, unsatisfactory sensitivity to change, and limited ability to identify underlying mechanisms eliciting impairments Paul et al. (2024); Vora et al. (2023). We are motivated to address these limitations in existing tactile examination methods by first comprehensively examining factors that could influence tactile perceptual outcomes in a population who is neurotypical (young adults), as well as aged (older adults). By testing for interaction effects between each of these factors that were previously not explored (e.g., arm, nerve, location), findings could potentially inform the future design of more effective tactile assessments.

Tactile perception is known to worsen with aging, leading to tactile impairments in older adults Correia et al. (2016). These tactile impairments correspond to poorer quality of life and decreased functional independence Kleinman and Brodzinsky (1978); Zhang et al. (2011). Identified reasons for why tactile impairments arise with aging include altered neurological, integumentary, cardiovascular, and/or endocrine function Charles et al. (2010); Lv et al. (2022); Ma et al. (2014); Thornbury and Mistretta (1981). Due to its significance on daily life, and overall indication of well-being, the factors influencing perception of tactile stimuli remain an area of ongoing exploration Correia et al. (2016); Cai et al. (2023); Amaied et al. (2015); Brodoehl et al. (2013); Norman et al. (2013); Abdouni et al. (2018); Stevens and Cruz (1996).

Prior work suggests that tactile perception can differ depending on the dominance of the arm examined, given the preferential use of one limb over the other depending on the task being performed Cai et al. (2023). The impact of arm dominance on tactile perception is of particular interest due to the number of daily activities requiring intact tactile perception and the coordinated use of both arms. This effect of arm dominance has been extensively studied in sensorimotor control literature Sainburg (2002, 2014); Dexheimer et al. (2024). Based on this prior work, we propose that the threshold at which tactile stimuli are detected may also differ depending on whether the dominant versus non-dominant arm is stimulated. However, existing literature exploring the role of arm dominance on tactile perception remains limited, with testing mainly conducted at single nerves and locations, and findings being inconsistent Cai et al. (2023); Greenspan and McGillis (1994); Ghent (1961); Özcan et al. (2004); Boles and Givens (2011). Here, we aim to explore how the dominance of one’s arm can influence conscious perception of electrotactile stimuli that are applied to the surface of one’s skin by examining multiple nerves and locations at each arm of individuals who are right-arm dominant.

When considering which nerves in the hand to examine, the median, ulnar, and radial nerves are of particular interest given that they each innervate unique and distinct regions that span across the arm, including complete coverage of the hand. Each of these nerves relays tactile information from the innervated cutaneous tissue to the brain’s higher centers for processing Patel and Varacallo (2024). This innervation forms dermatomes, or specific skin areas, each uniquely supplied by sensory fibers from a particular nerve. The median and ulnar nerves, which innervate the majority of the palmar and dorsal surfaces of the hand, have the greatest dermatomal distribution when compared to the radial nerve Dykes et al. (1997); Lee et al. (2008). Additionally, the median and ulnar nerves are situated closer to the surface of the hand whereas the radial nerve is located deeper below the skin surface. For these reasons, we can expect differences in tactile assessment outcomes depending on the nerve examined. Notably, impairments in tactile perception can originate at individual nerves due to a variety of factors unrelated to the condition being tested, including undiagnosed conditions. However, much of the existing literature on tactile perception relies on findings from assessments focused on a single nerve, typically the median nerve Cai et al. (2023); Greenspan and McGillis (1994); Ghent (1961); Özcan et al. (2004). We propose that examining multiple nerves is a safeguard to avoid misinterpretation based on single nerve(s), as well as of interest for presenting a complete picture of tactile function at the hand.

Another consideration for tactile assessments is how tactile stimuli are perceived depending on the location of the arm stimulated. Intact tactile perception at each joint, extending along the entire length of the arm, is critical for functional arm use Patel and Varacallo (2024). Accordingly, prior studies and clinical practice have investigated tactile perception by examining outcomes at differing arm locations. Their findings suggest that differences can arise in tactile perception depending on the arm location stimulated (e.g., hand versus elbow) Paul et al. (2024); Cai et al. (2023); Post et al. (1994); Lincoln et al. (1998). However, this approach of testing multiple arm locations when examining tactile perception is not always performed, including when investigating which factors impact tactile perception in individuals who have experienced disruption to the nervous system Paul et al. (2024); Ghent (1961); Özcan et al. (2004); Boles and Givens (2011); Glover et al. (2024).We propose that incorporating assessments at various locations on the arm ensures a more accurate understanding of tactile perceptual function.

Another important consideration when investigating tactile perception at the upper extremity is sex. Prior work identifies an effect of sex on tactile perceptual outcomes, with the effect depending on one’s age Abdouni et al. (2018); Peters et al. (2009); Bikah et al. (2008); Woodward (1993); Tan and Tan (1995); Kim et al. (2011). In young adults, females were found to have lower tactile detection thresholds in comparison with males Peters et al. (2009); Bikah et al. (2008); Woodward (1993); Tan and Tan (1995). However, when older adults were examined, findings of whether sex has an effect became conflicted Abdouni et al. (2018); Kim et al. (2011).

In this study, we aim to better understand the impact of arm dominance, nerve, location, and sex on tactile perception in young adults and older adults. To address this aim, we examined the role of each of these factors, including their previously unexplored interactions, on the conscious perception of electrotactile stimuli that are applied to one’s skin. We determined detection thresholds, defined here as the minimum intensity at which electrotactile stimuli applied to one’s skin become consciously perceptible. These thresholds were measured at the median, ulnar, and radial nerves, testing both the hand and elbow of each arm in right-arm dominant males and females who are young and older. By systematically measuring these detection thresholds, we could assess how detection of electrotactile stimuli applied to one’s skin varies according to arm, nerve, location, and sex. Based on this testing, we provide normative results surrounding how each of the factors, and their interactions, influence perception of electrotactile stimuli applied to the skin of each arm. These findings can inform the future design and implementation of potentially more reliable electrotactile assessments that capture variability due to each of these factors prior to interpretation. More broadly, insights from this study could also inform the design and optimization of electrotactile stimulation methods for additional applications including haptic feedback devices, sensory substitution systems, and assistive technologies Ray et al. (2024, 2021); Kaczmarek et al. (1991).

## Results

Here, we present the results summarizing participants’ ability to detect electrotactile stimuli applied to the skin when at the different nerves (median, ulnar, and radial) and distinct locations (elbow, hand; dominant arm, non-dominant arm) are stimulated.

**Fig. 1:**
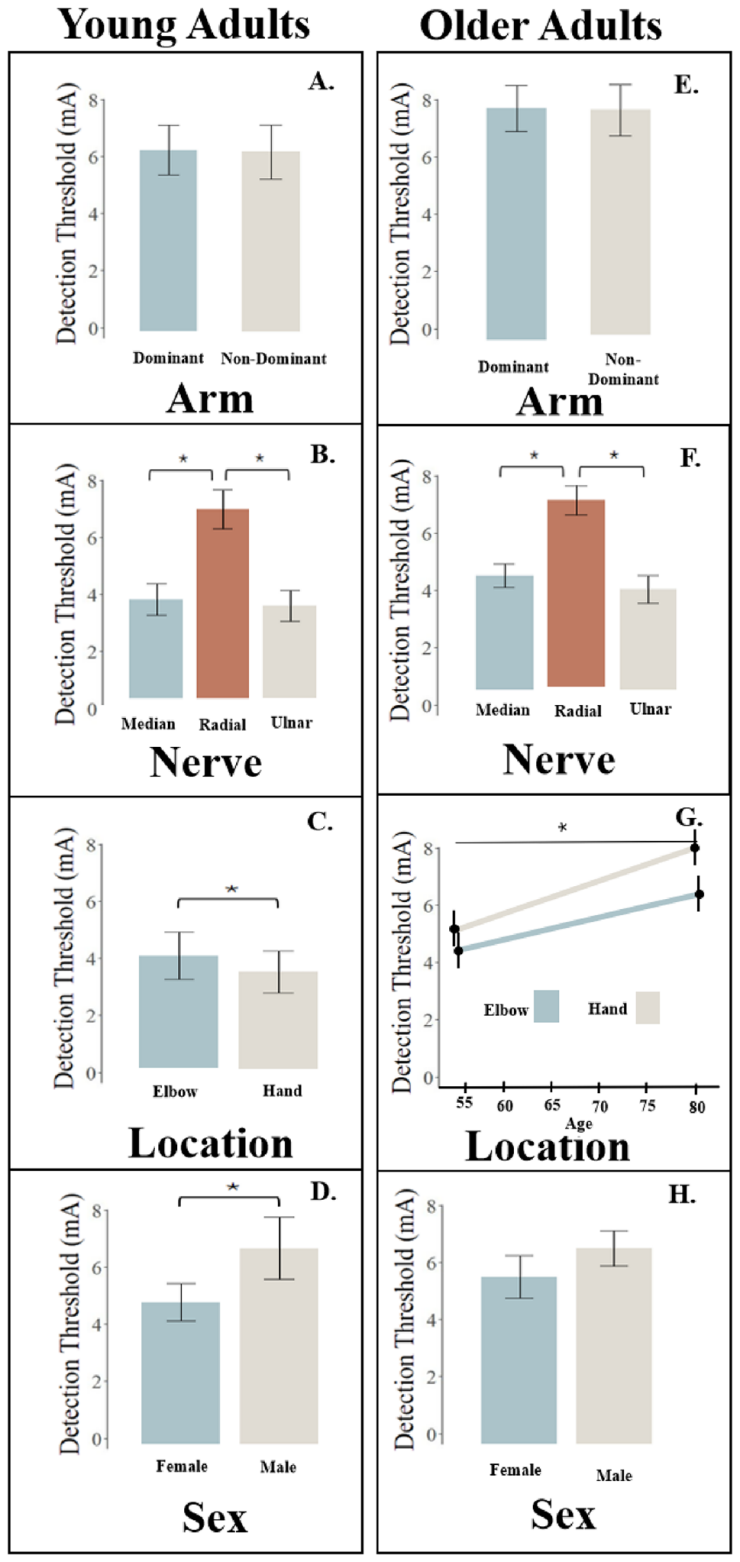
Role of Arm Dominance, Nerve, Location, and Sex on Detecting Electrotactile Stimuli Applied to the Skin in Young Adults and Older Adults. The bar plots indicate the mean (bar height) and standard deviation (error bars) of the detection thresholds for 20 young adults and 14 older adults who are all right-arm dominant. Statistical significance is indicated by a line with a star above. Plots A-D provide outcomes for the young adults, while plots E-H provide outcomes for the older adults. Plot G illustrates the interactive relationship between age and location, showing how their combined effects significantly influence detection. The different manner by which the data are presented in Panel G is because only these data for the older adults had the interaction effect

### Young adults

Results for the young adults are summarized in plots A-D of Fig. 1. The detection threshold did not statistically significantly differ depending on the dominance of the arm examined for the tested right-arm dominant participants (F(1,176) = 0.17, p = 0.678). In contrast, nerve had an effect on the detection threshold (F(2,176) = 233.91, p<0.001), with the radial nerve (7.30 mA) having a higher detection threshold than the median nerve (3.83 mA) and ulnar nerve (3.59 mA). When analyzing the location stimulated, the detection threshold at the elbow (3.92 mA) was higher than at the hand (3.49 mA) (F(1,176) = 9.01, p = 0.003). The detection threshold differed depending on sex (F(1,18) = 19.11, p<0.001), with the males (5.38 mA) having a higher detection threshold than the females (3.92 mA). We included age in the statistical model and observed no statistically significant effect on the detection threshold (F(1,18) = 1.86, p = 0.190). Combined, these results indicate that thresholds at which electrotactile stimuli applied to the skin are detected differ depending on the nerve, arm testing location, and sex in young adults. Yet, these findings did not support our initial hypothesis that the detection threshold would significantly differ depending on the dominance of the arm tested in our right-arm dominant participants.

### Older adults

Results for the older adults are summarized in plots E-H of Fig. 1. Arm dominance was not found to statistically significantly affect the detection threshold in our right-arm dominant participants (F(1,813) = 1.18, p = 0.279). Nerve did have an effect on the detection threshold (F(2,813) = 586.56, p<0.001), with the radial nerve (8.67 mA) having a greater detection threshold than the median nerve (5.21 mA) and ulnar nerve (4.59 mA). An interaction effect between age and arm location was observed on the detection threshold (F(1,812)=68.16, p<0.001); the detection threshold increased with age to a greater extent at the hand than at the elbow. At the age of 55, the estimated detection threshold was 5.05 mA at the hand and 4.77 mA at the elbow. At the age of 80, the estimated detection threshold was 8.00 mA at the hand and 6.43 mA at the elbow. Sex was not found to have a statistically significant effect on the detection threshold (F(1,12) = 2.72, p = 0.125). Additionally, body-mass index (BMI) (F(1,11) = 1.35, p = 0.269) and temperature (F(1,812) = 2.14, p = 0.144) were not found to have a statistically significant effect on the detection threshold. Combined, these results indicate that the threshold for detecting electrotactile stimuli applied to the skin differed depending on the nerve. Moreover, these results indicate that the negative impact of aging on tactile detection is greater at the hand than at the elbow. However, similar to our finding in young adults, the results did not reveal an impact of the dominance of the arm tested on the detection threshold in our right-arm dominant participants. These findings suggest that tactile perception can vary within each arm, and that this perception is susceptible to decline with aging, which may be more severe distally.

### Comparing young adults and older adults

The between-groups statistical analyses revealed that young adults detected the electrotactile stimuli at thresholds that were, on average, 0.99 mA less than the older adults (F(1, 1502) = 136.70, p < 0.001). The amplitude at which the electrotactile stimuli were just detected at the non-dominant arm was, on average, 0.13 mA less than at the dominant arm (F(1, 1502) = 5.30, p = 0.021). Nerve did had an effect on the detection threshold (F(1, 1502) = 840.02, p < 0.001), with the radial nerve having a greater detection threshold by 3.47 mA than the median nerve and ulnar nerve. We found a significant interaction between age group and location (F(1, 1501) = 159.69, p < 0.001), where the extent to which the detection threshold increased from young adults to older adults was greater at the hand than at the elbow. In the young adults, the estimated detection threshold was 4.2 mA at the hand and 5 mA at the elbow. In the older adults, the estimated detection threshold was 5.4 mA at the hand and 5.6 mA at the elbow. Additionally, we found a significant interaction between sex and age group (F(1, 1502) = 16.22, p < 0.001), where females had a greater increase in the detection threshold with age than males. In the young adults, the estimated detection threshold was 3.8 mA in the female participants and 5.4 mA in the male participants. In the older adults, the estimated detection threshold was 5 mA in the female participants and 5.6 mA in the male participants. Given that BMI was not obtained for the young adults, BMI was excluded from this between-groups analysis and, hence, is not reported. Combined, these results indicate that electrotactile detection thresholds vary depending on the age group assessed. These findings reinforce that tactile perception declines with age, and that this decline may be more pronounced in distal regions of the arm. Additionally, the findings suggest a greater decline in consciously detecting tactile stimuli with age in females than in males.

## Methods

### Human subjects study

This study received approval from the Virginia Tech Institutional Review Board (23-1246). For this study, sex was defined as male or female based on the binary sex categorization assigned at birth. Additionally, gender identity, which may differ from sex assigned at birth, was recognized, and categories such as male, female, transgender, and non-conforming/non-binary were included. Demographic data were collected in accordance with the 2024 National Institutes of Health guidelines.

#### Eligibility criteria

All participants were required to be right-arm dominant and have no neurological injuries and no musculoskeletal injuries at the upper extremities. In the young adult population, eligibility requirements included being between 18 and 23 years old. This age range was selected given the goal of characterizing typical behavior when processes governing development have largely peaked (in children) and processes inducing decline have largely not begun (in older adults) Bonnie et al. (2015). Eligibility requirements in the older adult population included being 55 years or older given prior work indicating that tactile perception can start to deteriorate at this age Thornbury and Mistretta (1981); Kemp et al. (2014).

#### Study participants

The study included 20 young adults [7 males, 13 females; aged (median, lower quartile, upper quartile, range): 22, 20, 22, 18–23 years] and 14 older adults [7 males, 7 females; aged (median, lower quartile, upper quartile, range): 66, 59, 72, 56–79 years]. These sample sizes for the young adults and older adults were each selected based on power analyses that we conducted. We point out that 15 older adults consented to participate, with one participant withdrawing from the study due to previously suggested externally-related concerns; no data were collected from this participant beyond the initial consenting process. All participants were right-arm dominant, and their sex and gender identity matched.

**Fig. 2:**
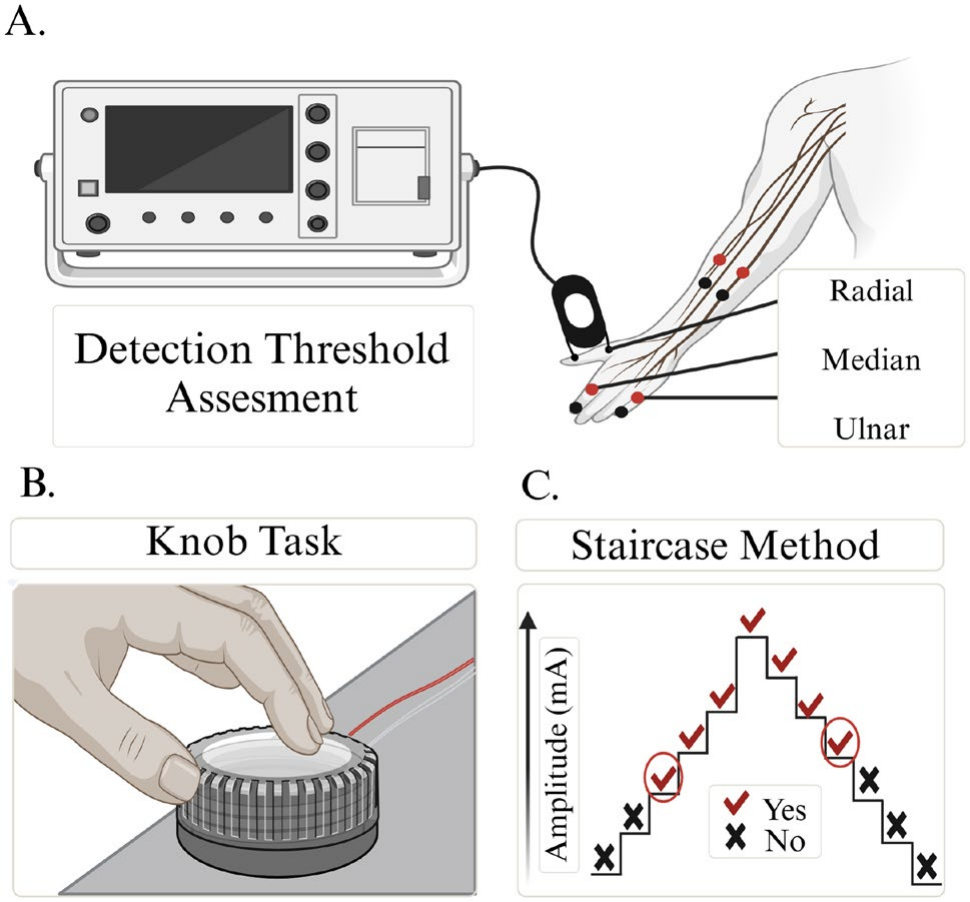
Experimental Design and Electrode Placement. A Surface electrodes were positioned at digit 2 (skin innervated by the median nerve), digit 5 (skin innervated by the ulnar nerve), and the snuff box (skin innervated by the radial nerve) for hand stimulation. Stimulating electrodes were positioned at the skin innervated by the median and ulnar nerves of the elbow (1 cm distal from the elbow crease and 4 cm proximal from the ulnar side of the arm, respectively) for elbow stimulation. The cathode electrode was placed 3 cm distal to the anode surface electrode for all testing conditions.^1^ B This schematic illustrates the knob task, as participants were asked to turn a physical knob, which, in turn, controlled the stimulus amplitude. C The schematic illustrates the staircase procedure. The symbols ‘$\checkmark$’ and ‘$\times$’ represent when the participants were able or not able to detect the electrotactile stimulus, respectively. Encircled responses highlight the first time the participants indicated they could perceive the stimulus on the upward staircase and the final time the participants indicated they could perceive the stimulus on the downward staircase. These encircled responses were used to determine the detection threshold. ^1^This image was created with BioRender.com.

### Experimental setup

A commercial device (DIGITIMER, Model: DS8R; Welwyn Garden City, England) delivered electrotactile stimuli to the surface of the skin that was innervated by each targeted nerve of each hand and elbow. An electrotactile stimulus consisted of a 100 $\upmu$s monophasic pulse, with its amplitude in the range of 0–100 mA given the goal of targeting the tactile A$\mathrm {\beta }$ fibers Canning (2020); Alon et al. (1983). As shown in Fig. 2, surface electrodes were positioned on the skin innervated by the median, ulnar, and radial nerves for hand stimulation, and on the skin innervated by the median and ulnar nerves for elbow stimulation. The radial nerve was not assessed at the elbow as subcutaneous needling is required due to the anatomy of the arm.

### Tactile assessments

All experimental data were captured at a sampling rate of 1 kHz. Data captured included electrotactile stimulus magnitude (mA) and time.

#### Knob adjustment assessment

To keep the duration of testing comparable among participants, the threshold at which the electrotactile stimulus could be just consciously detected was initially estimated using the method of adjustments (knob adjustment assessment). As depicted in Fig. 2B, the participants were instructed to rotate a knob clockwise and counter-clockwise, which respectively increased and decreased the electrotactile stimulus amplitude, until they just detected the electrotactile stimulus. The electrotactile stimulus was automatically delivered every 1 s such that participants were required to indicate every 1 s if they could detect the 100 $\upmu$s electrotactile stimulus pulse that was delivered. From this initial estimated threshold of when the electrotactile stimulus was just detected, the longer duration staircase procedure began to more precisely quantify the detection threshold Gescheider (1997); Kingdom and Prins (2016).

#### Staircase assessment

During the staircase procedure, each electrotactile stimulus (100 $\upmu$s pulse) was administered at randomized time intervals sampled between a 3–5 s distribution to prevent participants’ anticipation of the stimulus. Starting at 1 mA below the estimated detection threshold (based on the knob adjustment assessment), the magnitude of the electrotactile stimulus increased in an upward staircase. Similar to *Cai et al. (2023)*, stimuli were delivered in increasing intervals of 0.2 mA until participants verbally indicated three times in a row that the electrotactile stimulus was detected Cai et al. (2023). This marked the completion of one upward staircase. Then, the stimulus increased by 0.4 mA and continued in the opposite direction, beginning a downward staircase. Stimuli were delivered in decreasing intervals of 0.2 mA until the participants failed to detect the electrotactile stimulus three times in a row. This marked the completion of a downward staircase. Subsequently, the stimulus decreased by 0.4 mA before the upwards staircase repeated again. Every time the participants verbally confirmed perceiving the electrotactile stimulus, the experimenter pressed a button to record the amplitude of the electrotactile stimulus delivered and detected.

As indicated in Fig. 2C, data of interest included the first confirmation of the three consecutive verbal indications that the participants could perceive the electrotactile stimulus on the upward staircase and the final confirmation that the participants could perceive the electrotactile stimulus prior to the three consecutive failures on the downward staircase. Each upward and downward staircase was completed three times, producing six total values for when participants confirmed detecting the electrotactile stimuli. The detection threshold was determined as the average of these six values when confirming detection of the electrotactile stimulus.

### Experimental protocol

Participants were instructed to abstain from caffeine and other non-essential medications on the day of each session to avoid potential effects stemming from physiological alterations to nerve signal transmission Ward et al. (1981); Bättig et al. (1984). Upon obtaining the participants’ consent to take part in the study, the experimental protocol began and lasted approximately two hours.

To ensure typical cognitive and sensorimotor function in the older adults, several screening assessments were initially administered with this population. The participants completed the Montreal Cognitive Assessment (MoCA), allowing for screening of cognitive function Hoops et al. (2009). Scores of 26 or higher led to inclusion as these scores are indicative of being typical Carson et al. (2018); the maximum score possible is 30. Following, intact tactile perception was determined using Von Frey monofilaments Godde et al. (2018). Aligned with previously described methods, a monofilament was used to apply a force of 2.00 gms to the anatomical snuffbox (thumb – radial nerve), digit 2 (index fingertip – median nerve), and digit 5 (pinky fingertip – ulnar nerve) to ensure participants could detect the tactile stimulus and, accordingly, had intact protective sensation Kang et al. (2022). Additionally, two-point discrimination testing was performed at the same locations to further evaluate tactile function with a perceptual task that involves more cortical processing (discrimination task) than occurs with a detection task. Participants were required to score within the typical range (< 6 mm) on the two-point discrimination task to continue with their participation Lundborg and Rosén (2004). Finally, the nine-hole peg test was administered to screen for fine motor coordination and ability to follow simple directions. Participant scores were compared with normative data Feys et al. (2017), and performance within these norms was deemed acceptable. Additionally, BMI and body temperature data were collected in the older adults as this interest arose after data collection was completed in the young adults.

Next, our custom tactile examination experimental protocol began. Initially, participants underwent a training protocol during which they were taught what the electrotactile stimuli felt like and how to perform the experimental tasks (i.e., how to turn a knob for the knob adjustment assessment and how to complete one upward and downward staircase for the staircase assessment, as described in Sects. 3.3.1 and 3.3.2, respectively). This training was done to ensure participants understood the testing procedures and completed learning. Once participants indicated feeling comfortable with the procedures, testing began. The participants lay supine in a clinical bed and wore a blindfold and noise-canceling headphones to prevent distraction due to audiovisual stimuli Wesslein et al. (2014); Tipper et al. (2001). Data were collected one nerve and one location at a time. After completion of testing at one arm, the procedures were conducted at the opposite arm. The testing conditions were pseudo-randomized using a Latin square design, which determined the sequence of nerve, location, and arm presented.

### Statistical analysis

Initially, the data for the young adults and older adults were analyzed separately to capture effects arising within each group, independently. These data were fit to a linear mixed-effects model, with the fixed effects of testing arm, nerve, location, sex, and age, along with their interactions. In secondary analyses for the older adults, we included BMI and body temperature as fixed effects in the model to explore their potential impacts. Next, we captured differences between the young adults and older adults by analyzing all the data collected all together, with age group (young adult, older adult) as a factor and age removed as a factor. For each of these analyses, participant was included as a random effect and detection threshold as the dependent variable. An analysis of variance (ANOVA) identified significant interactive and fixed effects. The ANOVA was conducted using a hierarchical approach in which non-significant interaction terms were first removed, followed by non-significant main effect terms. Post-hoc pairwise comparisons, adjusted with a Tukey correction, determined which pairs significantly differed for a significant effect.

## Discussion

Tactile, or touch-related, perception is crucial for successful performance of daily tasks, like buttoning a shirt or holding a cup Cole (1992); Bolognini et al. (2016). While the critical role of intact tactile perception on functional independence is well-established, the underlying mechanisms driving tactile impairments often remain insufficiently understood, including in populations recovering from neurological injuries Paul et al. (2024); Vora et al. (2023). Previous studies have highlighted that this gap in understanding is often attributed to limitations in existing tactile assessment methods Paul et al. (2024); Vora et al. (2023). Here, we aimed to address this gap by first exploring different factors, and their interactions, that may influence tactile perception in a population that is neurologically intact. Existing literature indicates that the factors of arm dominance, nerve, location, and sex can influence varying aspects of sensorimotor control independently. Yet, when assessing tactile perception, prior literature does not account for all of these factors combined, including their interactions Cai et al. (2023); Greenspan and McGillis (1994); Ghent (1961).

In our study, we aimed to address this limitation by investigating if the threshold for detecting electrotactile stimuli applied to the skin generalizes across multiple nerves and locations of the dominant and non-dominant arms of right-arm dominant male and female young adults and older adults. These results can be used as normative outcomes for subsequent studies that investigate why tactile impairments arise in patient populations. Based on this knowledge, we anticipate that future work can identify how to more accurately examine tactile impairments in patient populations, including those following a neurological injury. Moreover, findings from this work could inform the design of electrotactile stimulators for use in scenarios such as haptic communication, sensory substitution, and assistive technologies Zhou et al. (2022); Visell (2009); Kaczmarek et al. (1991); Chouvardas et al. (2008).

### Role of arm dominance on tactile perception

Prior work indicates hemispheric lateralization, where each hemisphere specializes in differing aspects of a sensorimotor process Sainburg (2002, 2014), could contribute to sensorimotor differences depending on the dominance of the arm being used. Aligned with this notion, some prior studies Özcan et al. (2004); Boles and Givens (2011) did not find an effect of arm dominance, while others did Cai et al. (2023); Greenspan and McGillis (1994); Ghent (1961). In this work, we did not observe a significant impact of whether the dominant versus non-dominant arm was stimulated on the detection threshold when analyzing the data independently for the young adults and older adults who were all right-arm dominant. However, we did observe significantly smaller detection thresholds when the non-dominant arm was stimulated than the dominant arm when all the data were grouped together to compare the young adults to the older adults. The increased number of participants (34 in total) allowed for an effect to be observed, albeit for a relatively small effect size (0.13 mA difference).

A possible reason for not observing a significant effect when analyzing the data for the young adults and older adults in isolation is the simplicity of the task, as literature indicates that more complex tasks are needed to observe an effect of arm dominance on tactile perception Cai et al. (2023); Boles and Givens (2011); Liang et al. (2019); Casado-Palacios et al. (2023); Shimizu et al. (2023); Kilteni and Ehrsson (2022). For our protocol, participants were instructed to remain relaxed (i.e., no muscle activation) and indicate when an externally applied electrotactile stimulus was detected. Future work could use our comprehensive approach of testing multiple nerves and locations to determine whether the impact of the dominance of the arm on tactile perception generalizes across the arm during more complex tasks (e.g., motor activation task, cognitive-perceptual integration task).

Another possible reason for conflicting results regarding an impact of arm dominance on tactile perception is the lack of conformity in how tactile stimuli are provided. Existing literature reports findings based on stimulation using Von Frey monofilaments and electrotactile stimuli Paul et al. (2024); Vora et al. (2023). While limitations are known for each of these tactile stimulation approaches, these approaches are identified as the best currently available options for tactile examination Vora et al. (2023). Von Frey monofilaments involve testing with a small, fixed number of physical stimuli magnitudes (e.g., 6, 20), which limits its sensitivity, or ability to detect subtle changes in tactile perception. For example, participants with a very low or very high tactile detection threshold may not show measurable differences, highlighting the limitations in capturing nuanced changes in perception. Additionally, examinations with Von Frey monofilaments have conflicting findings in test-retest reliability, with some protocols demonstrating good reliability while others reporting poor reliability Tracey et al. (2012); Paul et al. (2024). Differences in reliability may stem from variability in stimulus application, including timing, force, and location Tracey et al. (2012). While certain methodologies optimize reliability, the potential for examiner-dependent variability remains a concern, particularly in studies requiring precise threshold measurements. In considering electrotactile stimulation, this approach has great test-retest reliability Zhou et al. (2022, 2023). One reason for this great test-retest reliability is its sensitivity to capture very small changes due to the ability of delivering precise stimuli repeatably across a very large range of stimuli. Another reason is that variability from the assessor during the examination can be avoided by automating the timing, location, and magnitude when applying the electrotactile stimuli. Despite these benefits, perceptual outcomes can differ between tested locations when using electrotactile stimuli for reasons including the positioning of the electrodes and the preparation of the skin Zhou et al. (2022, 2023). Therefore, an impact of the experimental setup, rather than human perception, could lead to differences in perceptual outcomes depending on the tested location. Future work could consider standardized protocols to minimize these inconsistencies and enhance comparability across studies; this study did use a standardized protocol to limit such potential effects. We recommend testing multiple locations on each arm to help reduce this potential undesired experimenter-induced variability, regardless of the tactile assessment approach and standardization protocol used.

### Role of nerve on tactile perception

The threshold for detecting electrotactile stimuli applied to the skin was higher at the radial nerve than the ulnar nerve and median nerve in both arms of the young adults and older adults when analyzing the data independently. This trend remained the same when analyzing the data grouped all together for the young adults and older adults. This distinction in the detection threshold between nerves could be attributed to anatomical differences at the upper limb. The palmar aspect of the hand (innervated by the median and ulnar nerves) has greater receptor densities than the dorsal aspect of the hand (innervated by the radial nerve) Mitchell and Whited (2024); Hernández-Cortés et al. (2022). It is reasonable to expect that the applied electrotactile stimuli activate more nerve fibers innervating the palmar aspect than the dorsal aspect of the hand, leading to improved perception. Another consideration is that a stronger electrotactile stimulus may be required to activate the radial nerve than the ulnar and median nerves since the radial nerve is situated deeper below the skin Tommerdahl et al. (2010). We point out that since the surface electrodes applied electrotactile stimuli to the skin surface, the superficial nerve fibers near the skin were targeted. Intraneural stimulation could access deeper nerve bundles, which could potentially lead to differing results. All together, we propose that our findings regarding an impact of nerve on thresholds for detecting electrotactile stimuli applied to the skin reflect anatomical differences, including variations in receptor density and nerve depth Hernández-Cortés et al. (2022).

### Role of arm location on tactile perception

We observed that the location on the arm where the electrotactile stimuli were applied impacted the threshold at which the electrotactile stimuli could be detected in both the young adults and older adults. When the data were analyzed all together, there was a significant interaction between age group and location, with the extent to which the detection threshold increased from young adults to older adults being greater at the hand than at the elbow. One reason that tactile perception may differ across age groups, particularly in distal regions such as the hands, could be due to age-related physiological changes Correia et al. (2016); Metzner et al. (2022); Brodoehl et al. (2013); Decorps et al. (2014). These changes are further explored in Sect. 4.3.2.

#### Young adults

In the young adult population, when their data were analyzed independently, we observed that the threshold for detecting the electrotactile stimuli applied to the skin was lower at the hand than at the elbow. This finding corroborates prior work demonstrating that the hand is more sensitive in detecting tactile stimuli than other parts of the arm Post et al. (1994); Chapman (1994); Caruso et al. (1994)Ȧn explanation for why the hand is more sensitive than the elbow is the density of innervation is greater at the hand than at the elbow Mitchell and Whited (2024); Johansson (1978); Corniani and Saal (2020). In turn, we can expect that the applied electrotactile stimuli will have a greater chance at activating more nerve fibers innervating the hand than the elbow. Another important anatomical factor is that the nerves at the elbow are located deeper beneath the skin compared to those at the hand Tommerdahl et al. (2010). As a result, a stronger electrotactile stimulus is needed to activate these deeper-lying nerves, due to the greater physical distance between the nerve and the electrodes. From an evolutionary perspective, humans have relied heavily on their hands for survival when compared to the elbow, further supporting the need for greater innervation at the hand. Consequently, our finding that the hand is more sensitive to tactile stimuli than the elbow aligns with the findings of prior studies.

#### Older adults

In contrast to the findings in the young adults, we observed that detection thresholds were, on average, higher at the hand compared to the elbow in older adults when their data were analyzed independently. Additionally, we observed a significant interaction between the location stimulated and the age of the participant, such that thresholds at the hand increased to a greater extent than at the elbow with increasing age. To the best of our understanding, this age-related impact is not currently known. This pattern may reflect age-related physiological changes that occur across systems such as the nervous, circulatory, integumentary, and endocrine systems Charles et al. (2010); Lv et al. (2022); Ma et al. (2014); Thornbury and Mistretta (1981). Age-related changes within these systems can contribute to poorer tactile perception Correia et al. (2016), whereas such age-related changes are not observed in young adults who typically maintain intact physiological function and tactile sensitivity during this stage of their lives Adamo et al. (2007); Amaied et al. (2015); Brodoehl et al. (2013); Metzner et al. (2022); Decorps et al. (2014). Specifically, the distal regions of the body, such as the hands, are more vulnerable to age-related changes, including peripheral nerve degeneration, reduced capillary density, and compromised skin integrity Metzner et al. (2022). These changes may more severely impair signal transmission and tactile sensitivity at distal sites, aligning with literature suggesting that tactile impairments often emerge and are more pronounced distally than proximally, including in an aging population Metzner et al. (2022); Samain-Aupic et al. (2024).

### Role of sex on tactile perception

The threshold at which the electrotactile stimuli applied to the skin could be detected differed depending on one’s sex in young adults, but not in older adults, when their data were each analyzed independently. When the data were grouped all together, the analysis revealed an interaction effect between age group and sex, with the detection threshold increasing to a greater extent in females than males with age. Combined, these findings suggest that while sex differences in tactile perception may be evident at a younger age, these differences may diminish with age.

#### Young adults

The threshold at which the electrotactile stimuli applied to the skin could be detected differed depending on sex, with females having a lower detection threshold when compared to males. Sex differences in tactile perception are often attributed to factors such as fingertip size, skin compliance, hormones, BMI, behavior socialization, and body temperature, which vary between males and females Boles and Givens (2011); Peters et al. (2009); Woodward (1993); Karastergiou et al. (2012); Kim et al. (1998); Zheng et al. (2017). In our study, we utilized electrotactile stimuli applied to the skin, which bypasses cutaneous mechanoreceptors to activate free nerve endings directly. This approach ruled out the influence of fingertip size and skin elasticity, putting the focus on other physiological factors including hormonal differences and body temperature.

As relevant to tactile perception, BMI positively correlates with detection thresholds; as BMI increases, it becomes more difficult to perceive tactile stimuli Navarrete-Muñoz et al. (2020). Based on this information, we would expect males to detect the electrotactile stimuli at higher amplitudes than the females since males typically have a higher BMI Karastergiou et al. (2012). The findings in our study supported this hypothesis, with males have a higher detection threshold than females. That said, we did not capture BMI in the young adult population, so we do not know if our participants reflected norms of males having a larger BMI, on average, than females.

Body temperature negatively correlates with detection threshold, such that increasing body temperature makes it possible to detect stimuli at lower amplitudes Kim et al. (1998); Todnem et al. (1989); Dioszeghy and Stålberg (1992). As relevant to sex, it has been reported that females have a higher body temperature than males Kim et al. (1998). Therefore, it is reasonable to expect that females can detect stimuli at lower amplitudes than males, which aligns with our findings. We did not track or control for body temperature and, hence, cannot indicate whether the differences in the detection threshold were temperature dependent.

Another factor to consider regarding the impact of sex on tactile perception is a potential behavioral socialization effect Zheng et al. (2017). Females and males may respond whether they detect tactile stimuli differently based on differing societal expectations, with one group more readily stating ‘yes’ than the other. Future work could determine whether differences in the manner by which females and males socialize correspond to differences in their responses during the tactile examination.

#### Older adults

Corroborating previous research Abdouni et al. (2018); Peters et al. (2009); Bikah et al. (2008); Woodward (1993); Tan and Tan (1995); Kim et al. (2011) in older adults, sex was not found to significantly affect tactile perception in our tested older adults. This finding in the older adults contrasts the significant effect of sex found in the younger adults. As discussed in Sect. 4.4.1, factors such as BMI and body temperature may impact tactile perception when utilizing electrotactile stimuli. Data were collected from the young adults prior to the older adults so we included BMI and body temperature measures to examine a broader range of influences on tactile perception in the older adult population. When analyzing the potential roles of BMI and body temperature on sex differences, we found that neither sex nor BMI showed a significant effect on tactile perception in this older population.

Another consideration is a sex-dependent change with aging. Studies comparing young adult males to older adult males show no significant differences in tactile perception Abdouni et al. (2018). However, when young adult females are compared to older adult females, significant changes in tactile perception are observed Abdouni et al. (2018). This result suggests that sex differences in tactile perception may be driven by factors present in young adult females, but not in older adult females. One potential explanation is that hormonal changes post-menopause may attenuate observed sex differences. Estrogen, which has been shown to play a neuroprotective role, declines with aging Kim et al. (2011); Singh et al. (2016); Shvetcov et al. (2023); Ustaömer et al. (2021). This decline in estrogen levels with aging might contribute to changes in peripheral nerve sensory function and overall tactile perception, contributing to the sex-dependent results we observed Tan and Tan (1995); Meador et al. (1998). Future work can explore such variables that could explain differences in detection thresholds depending on sex and age.

### Limitations

A limitation of this research is the exclusion of individuals who are left-arm dominant, despite research indicating that approximately 10$\%$ of the population is left-arm dominant de Kovel et al. (2019). Accordingly, it remains unclear whether the results obtained from this protocol in individuals who are right-arm dominant would generalize to individuals who are left-arm dominant.

An additional limitation is that we did not examine all nerves at all locations of the arm. Accordingly, our findings cannot be generalized to the entire arm. This limitation is a feasibility constraint since we needed to decide how many nerves and locations to examine to successfully complete this research and draw conclusions. We targeted the three main nerves at the hand, and tracked two of those nerves, which could be tested more proximally, at the elbow, with the goal of having a robust, while simultaneously practical approach for examining tactile perception. Future studies could be more ambitious in the scope of variables examined to gain an even more comprehensive understanding of the impact of differing factors, and their interactions, on tactile perception.

Another limitation is that we draw conclusions about tactile perception based on electrotactile stimuli. Electrotactile stimuli bypass mechanoreceptors whereas physically applied tactile stimuli activate the cutaneous mechanoreceptors. Even so, both electrotactile stimuli and physically applied tactile stimuli largely engage the same neural pathways (e.g., dorsal column medial lemniscal pathway), enabling relevant interpretations of tactile function and perception.

Finally, another limitation is that our conclusions are based on a relatively small sample size from southwest Virginia, which may not be generalizable to broader populations. We acknowledge that our sample of 20 young adults and 14 older adults, while sufficiently powered for this work, may not represent these populations as a whole. Future studies could extend this work by examining diverse populations, thereby enhancing the robustness and applicability of our results. Additionally, our testing scope was limited to young adults (18-23 years old) and older adults (55+ years old), which may influence the interpretation of our findings. Further research is needed to determine whether these effects persist across different age groups and demographics.

### Future considerations

The findings of this study emphasize the need to consider multiple variables and their interactions, including nerve, location, and sex, when designing tactile assessments for patient populations. By establishing normative data and pinpointing variables and their interactions that influence tactile perception, this work provides a foundation for developing more precise and personalized assessments. To determine whether the observed differences translate to meaningful changes in arm function, future research can determine the minimal clinically important differences for electrotactile stimuli in the context of tactile perception. Moreover, exploring how electrotactile perception compares to other forms of sensory stimulation, such as force or vibrotactile stimuli, could help contextualize these findings within broader efforts to further our understanding of tactile perception and its underlying mechanisms. Future work can also integrate these insights into clinical protocols to facilitate earlier identification and targeted interventions for tactile impairments, including in individuals with neurological injuries.

To expand beyond our study of electrotactile stimuli, future work can examine perception of tactile stimuli that physically interact with the skin (e.g., vibrotactile stimuli). Examining these physical interactions, along with their respective frequency-dependent effects Graczyk et al. (2022); Gurari et al. (2017), could provide deeper insight into sensory processing mechanisms that were beyond the scope of this work. Furthermore, future studies can consider the influence of sex-based behavioral biases on tactile perception, perhaps by testing individuals from different cultures who exhibit differing sex-based behavioral tendencies. Finally, future efforts could use our findings to inform the design of electrotactile displays for areas such as haptic feedback and sensory substitution systems.

## Conclusion

This study explored how a variety of factors (dominance of arm, nerve, location, sex), and their interactions, impact tactile perception by examining ability to consciously detect electrotactile stimuli that are applied to the skin in young and older males and females who are right-arm dominant. An effect of nerve, location, and sex on detecting tactile stimuli was found in the young adults, and an effect of nerve and an interaction between location and age was found in the older adults. Results obtained here could be used as normative data for comparison with future studies that aim at understanding underlying mechanisms that may elicit impaired tactile perception following a diagnosis, such as a neurological injury. Additionally, given the relevance of electrotactile displays for haptic technologies, our findings may offer valuable insights for the development of electrotactile stimulation methods for these applications.

## Data Availability

The data that support the findings of this study are available upon reasonable request.
